# Does Addition of Phosphate and Ammonium Nutrients Affect Microbial Activity in Froth Treatment Affected Tailings?

**DOI:** 10.3390/microorganisms9112224

**Published:** 2021-10-26

**Authors:** Juliana A. Ramsay, Mara R. de Lima e Silva, Michael A. R. Tawadrous, Bruce A. Ramsay

**Affiliations:** Chemical Engineering, Queen’s University, Kingston, ON K7L 3N6, Canada; mara.silva@queensu.ca (M.R.d.L.eS.); michael.tawadrous@queensu.ca (M.A.R.T.); bruce.ramsay@queensu.ca (B.A.R.)

**Keywords:** oilsands tailing ponds, mature fine tailings, ammonium, phosphate, microbial activity

## Abstract

We examined greenhouse gas (GHG) production upon the addition of ammonium and phosphate to mature fine tailing (MFT) samples from Alberta’s Pond 2/3 (at 5 and 15 m) and Pond 7 (12.5 m) in microcosm studies. The methane production rate in unamended Pond 2/3 MFT correlated with sample age; the production rate was higher in the less dense, more recently discharged MFT samples and lower in the denser, deeper sample. Adding small amounts of naphtha increased methane production, but there was no correlation with increasing naphtha, indicating that naphtha may partition into bitumen, reducing its bioavailability. Although non-detectable phosphate and low ammonium in the pore water indicate that these nutrients were potentially limiting microbial activity, their addition did not significantly affect methanogenesis but somewhat enhanced sulphate and nitrate reduction. Neither ammonium nor phosphate were detected in the pore water when added at low concentrations, but when added at high concentrations, 25–35% phosphate and 30–45% ammonium were lost. These ions likely sorbed to MFT minerals such as kaolinite, which have microbial activity governed by phosphate/ammonium desorption. Hence, multiple limitations affected microbial activity. Sulphate was less effective than nitrate was in inhibiting methanogenesis because H_2_S may be a less effective inhibitor than NO_x_^−^ intermediates are, and/or H_2_S may be more easily abiotically removed. With nitrate reduction, N_2_O, a potent GHG was produced but eventually metabolized.

## 1. Introduction

In 2021, Canada was ranked as having the 4th largest global crude oil reserves, amount to about 170,300 million barrels [1], and in 2020, it was the 4th largest global oil producer, producing 5.5 million barrels per day [2]. About 55% of Canada’s crude oil production is from Alberta’s oil sands. There are three major deposits (Peace River, Athabasca, and Cold Lake), which cover 142,200 km^2^, where ~3% can be surface mined [3], and this represents ~20% of Alberta’s oil sand reserves. The oil sand ore consists of 12% bitumen, 3–6% water, and the is rest mineral ore. Bitumen is separated from the ore through the Clark hot water process as an initial separation step. The oil recovered by flotation is subsequently purified by adding a hydrocarbon diluent (e.g., naphtha or paraffinic solvents) and by separating the remaining water and solids from the bitumen froth. The froth treatment tailings (FTT) are discarded to an oil sand tailings pond (OSTP). Although a majority of the diluent and bitumen are recovered, about 16 wt% solids, 1–2 wt% bitumen, and 0.2 wt% diluent (Per. Comm. E. Hollander, Suncor) [4] are discharged with the process water to the OSTP. With time, the tailings consolidate to form a dense suspension called mature fine tailings (MFT), where the older tailings at the bottom have a higher density of solids. The composition of the naphtha diluent varies with each company and includes low molecular weight C5 to C16 n-, iso-, and cyclo-alkanes as well as aromatic hydrocarbons such as benzene, toluene, ethylebenzene, and xylene (BTEX). OSTPs that receive FTT containing naphtha diluent typically have higher greenhouse gas (GHG) emissions such as CH_4_ and CO_2_, which arise from the microbial degradation of the biodegradable components of naphtha and bitumen under methanogenic conditions. Since CH_4_ has a global warming potential that is 28–36 times greater than CO_2_ over 100 years [5], understanding the factors that influence GHG production in OSTPs is important to develop strategies to minimize GHG production.

In most laboratory studies looking at GHG production, MFT samples were spiked with diluent or hydrocarbons and were frequently amended with a methanogenic medium such as the one used by Fedorak and Hrudey [6] and others [7,8,9,10,11,12,13,14] containing mineral salts such as NH_4_Cl, KH_2_PO_4_, and trace elements as well as vitamins. Although nutrients such as a nitrogen and/or phosphorous source can influence degradation rates, there have been no studies that have examined the impact of NH_4_^+^ and/or PO_4_^3^^−^ substrates on GHG production by the microbial population in MFT or examined potential interactions of NH_4_^+^ and/or PO_4_^3−^ with the minerals in MFT. There are many studies that have looked at the impact of NH_4_^+^ and PO_4_^3−^ on microbial activity, including hydrocarbon degradation activity, but we found very few specific to OSTPs or MFT. In one study, Collins et al. [15] found that an enrichment culture from an Albian MFT was able to fix nitrogen under methanogenic conditions, and this suggests that an ammonium source may not be needed. However, it is not known how such cultures fit into the MFT population. Furthermore, while there are many studies that have examined the interaction of NH_4_^+^ or PO_4_^3−^ in different soils and soil components, we once again did not find any published studies with respect to MFT. In this paper, we examined GHG production in unamended MFT samples, the impact of adding NH_4_^+^ and PO_4_^3−^ on GHG production under methanogenic, sulphate- and nitrate-reducing conditions, and the possible fate of that NH_4_^+^ and PO_4_^3−^. Three MFT samples were used: Suncor’s Pond 2/3 at 5 and 15 m and Pond 7 at 12.5 m. FTT is discharged to Pond 2/3, from which some MFT is transferred to Pond 7 for inventory management purposes. The water surface areas of Pond 2/3 and Pond 7 are 2.8 and 3.9 km^2^, respectively.

## 2. Materials and Methods

### 2.1. Chemicals

Monopotassium phosphate (KH_2_PO_4_) was obtained from Anachemia Chemicals Inc., Lachine, PQ, Canada, ammonium chloride (NH_4_Cl) was obtained from Fisher Scientific, Toronto, ON, Canada and naphtha was obtained from (Suncor Energy Inc., Edmonton, AB, Canada).

### 2.2. Description of MFT, Its Collection and Storage

The MFT samples were taken from Suncor’s Pond 2/3 at depths of 5 m and 15 m on 12 September 2018 as well as from Pond 7 at a depth 12.5 m on 10 September 2018. Tailings were stored in 20 L tightly sealed, high density polyethylene pails. Once the pails were received on 18 September 2018, the contents were stored at 2 °C until use.

### 2.3. Microcosm Set Up

Microcosms were set up in duplicate. Prior to use, the MFT was left at room temperature for about 24 h. To 60 mL of MFT in 125 mL microcosm bottles, 20 mL of sterile deionized water (unamended biotic controls) or 20 mL of sterile deionized water containing the appropriate concentration of phosphate and/or ammonium solution were added to a final liquid volume of 80 mL and a headspace of 45 mL. Microcosms were sealed with a butyl rubber stopper and an aluminum crimp (Fisher Scientific, Toronto, ON, Canada). The headspace was purged with ultra-high purity nitrogen gas (Praxair Canada Inc., Kingston, ON, Canada) for 30 min using a manifold system in the fume hood, according to the Hungate technique [16]. After purging, a slight positive pressure was maintained in each microcosm, and the initial pressure was measured, and the naphtha added. The surface of the butyl rubber stopper was made leak-tight by applying a few drops of 70% *v*/*v* ethanol and then burning off the alcohol. The contents of the microcosms were mixed in an orbital rotary incubator (Innova 44 series, New Brunswick Scientific, Enfield, CT, USA) at 200 rpm for 30–60 min; the mixing was repeated after every gas analysis, and the microcosms were incubated statically in the dark at room temperature (20 °C).

Abiotic controls were created by autoclaving the MFT with nutrient amendments as appropriate (not including naphtha) once per day for three consecutive days at 121 °C for 20 min [9] before sealing with butyl rubber stopper, purging with N_2_ gas, and adding naphtha.

### 2.4. Gas Analysis

The gas phase was analyzed using an Agilent 7890B gas chromatograph with a 1 mL fixed loop splitless injector connected to a PoraBOND Q column (50 m long, 0.53 mm diameter, and a 10 µm thick film, Agilent) and to an Agilent thermal conductivity detector. The pressure in the microcosms was measured with a digital pressure gauge (SSI Technologies, Inc., Janesville, WI, USA) prior to taking 5 mL of the headspace gas with a gas-tight Hamilton syringe attached to an 0.2 µm nylon syringe filter (25 mm diameter). Before sampling, the syringe and filter were flushed with N_2_ gas several times. After sampling the microcosm, a few drops of alcohol were placed on the septum and were flamed to seal any leaks. Dilutions of a calibration mixture of 6.5% carbon dioxide and 6.5% methane in nitrogen (Praxair Canada Inc.) was used to generate calibration curves, and the concentrations of CH_4_ and CO_2_ (µmol/100 mL MFT) were corrected for volume and headspace pressure.

### 2.5. Pore Water Analysis

#### 2.5.1. pH and Eh

Initial pH and Eh values were measured in sacrificial t = 0 microcosms. Once the experiments terminated, the final pH and Eh were measured in an anaerobic chamber (Nexus One Glove Box, Vacuum Atmospheres Company, Topsfield, MA, USA) using a pH electrode (LE409, Mettler Toledo, Columbus, OH, USA) and an Eh electrode (ORP InLab Redox Flow, Mettler Toledo, Columbus, OH, UAS) connected to a Mettler Toledo SevenEasy pH meter. An Orion™ ORP standard solution (Thermo Scientific, Toronto, ON, Canada) of Eh ~236 ± 6 mV was used to calibrate the ORP electrode.

#### 2.5.2. Water Chemistry

The pore water was collected from 30 mL of relatively homogenous MFT microcosm samples and was placed into 50 mL conical polypropylene centrifuge tubes (Fisher Scientific) and centrifuged at 8000 rev/min for 45 min [7] at 8 °C using a Sorvall RC-5B centrifuge. The recovered pore water samples were filtered to remove the oil phase, and any suspended fines were removed using a 5 mL gas-tight syringe (Model 1005, Hamilton Company, Reno, NV, USA) attached to a nylon syringe filter (0.2 µm pore size, 25 mm diameter, VWR, Toronto, ON, Canada).

Phosphate, sulphate, and nitrate were measured by the Analytical Services Unit, Queen’s University, using a Dionex HPLC system (ICS-3000) equipped with a Dionex AG4A-SC guard column and an AS4A-SC analytical column connected to a conductivity detector operated in a suppressed conductivity mode. A calibration curve of standards, which ranged from 0.08 to 50 mg/L, was used to quantify concentrations. Ammonium was determined by a colorimetric assay using a continuous flow AutoAnalyzer (QuAAtro AutoAnalyzer, SEAL Analytical, Inc., Mequon, WI, USA) in which the ammonium reacted with Berthelot’s reagent, which is an alkaline solution of phenoxide and sodium hypochlorite. The addition of sodium nitroprusside intensified the color, which was measured colorimetrically at 630 nm [17].

### 2.6. Total Dried Solids

The amount of MFT solids was measured according to Symons and Morey [18]. Each 20 L pail was mixed on a drive roll mixer (2″ diameter, 24″ long, 2-roll, Sepor Inc., Los Angeles, CA, USA) for 2 h. A volume of 16 mL of MFT was pumped from each bucket (Pond 7 and Pond 2/3 at 5 m or 15 m) and was placed on pre-weighed, clean, dry glass Petri dishes and dried at 105 °C overnight. The Petri dishes plus sample were cooled in a desiccator before re-weighing to constant weight.

### 2.7. X-ray Diffraction

Ammonium and phosphate were mixed with MFT for 30 min and were then left to equilibrate at room temperature for 24 h. About 10 g of this mixture was placed on a glass Petri dish and was allowed to dry in the fume hood for 24 h. The samples were disaggregated with a mortar and pestle before ~5 g were analyzed by X-ray diffraction (XRD). XRD was performed with an X-ray diffractometer (X’Pert Pro MPD, PANalytical, Henderson, NV, USA) with Co-Kα radiation (1.78 Å) operating at 40 kV and 45 mA. The 2θ angle was varied from 5° to 90° with a scan X’Celerator detector of 90 s/step.

## 3. Results and Discussion

### 3.1. Characterization of MFT

Pond 7 (12.5 m) had the lowest amount of total dried solids followed by Pond 2/3 at 5 m and then Pond 2/3 at 15 m (Table 1). This reflects the relative age of the MFT samples since discharge. Because the amount of solids increases with the settling time, it can be inferred that the Pond 7 MFT samples had the shortest consolidation times and that the 15 m depth samples from Pond 2/3 had the longest consolidation times. Pond 2/3 had higher levels of ammonium and sulphate at 15 m (35.3 and 100 mg/L, respectively) than at 5 m (20.8 and 20 mg/L, respectively), while Pond 7 had no detectable sulphate and a much lower level of ammonium (6.4 mg/L). No detectable amounts of phosphate, nitrate, or nitrite (Table 1) were found in the pore water of any pond sample. The lack of detectable phosphate suggests that biodegradation rates may be limited by phosphate availability. Low ammonium levels may also play a role. These data support the need to understand the effect of PO_4_^−3^ and/or NH_4_^+^ on the microbial activity in MFT samples.

### 3.2. Methanogenic Metabolism

**Figure 1:**
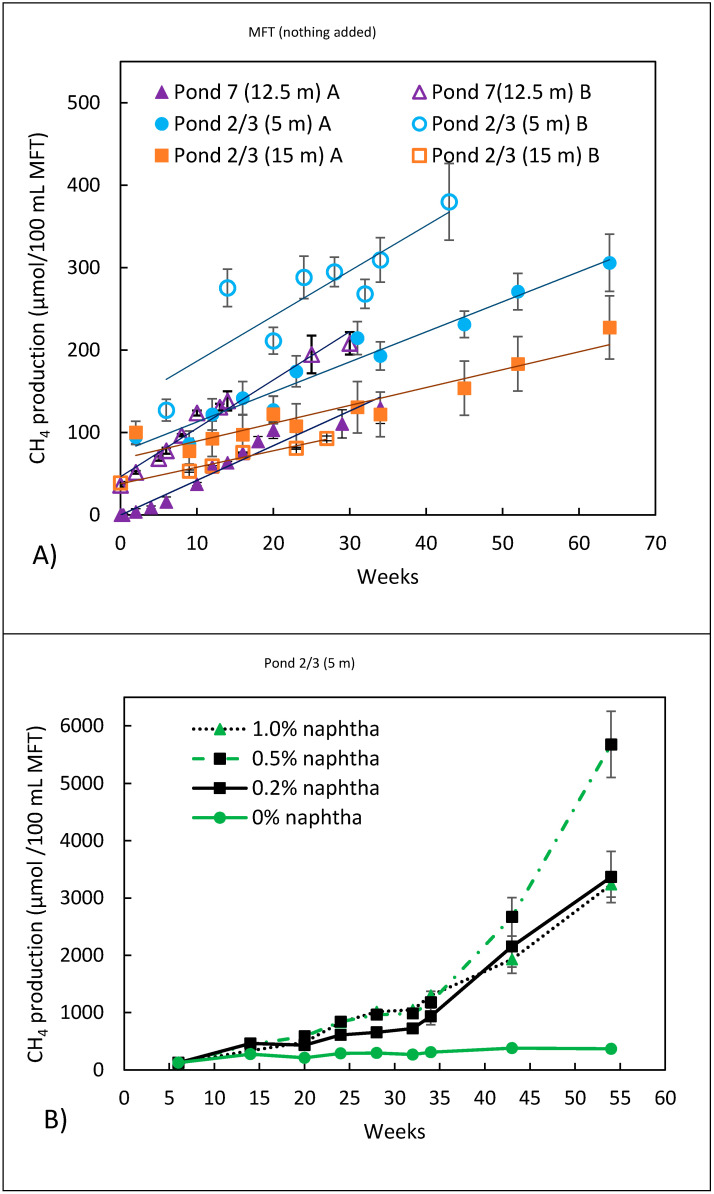
(**A**) Methane production of duplicate microcosms for each MFT sample in two independent experiments, each performed in duplicate. No amendments were added. The solid line represents the average rate of methane production for each pond sample as baseline conditions. (**B**) Methane production from duplicate MFT samples from Pond 2/3 at 5 m when naphtha alone was added. After naphtha addition, microcosms were mixed for 30–60 min on a rotary shaker then incubated in the dark. Error bars have been inserted, and where not visible, the error was too small to be seen.

Amended or unamended abiotic controls had no biological activity, i.e., no detectable methane production. Unamended biotic controls (i.e., no additives) had low but statistically significant biological activity, which was determined using one-way ANOVA with Dunn’s post hoc test (*p* < 0.005 for any unamended sample vs. abiotic control). Figure 1A shows a plot of two independent experiments performed in duplicate for each MFT sample incubated in the dark without amendments. The average rate of CH_4_ production for each sample (Table 2) represents baseline conditions in the tailing ponds, which were all methanogenic, even though Pond 2/3 at 5 m and at 15 m had 20 and 100 mg/L of sulphate, respectively. Methane production has been previously reported with ≤20 mg/L sulphate in MFT from the Mildred Lake Settling Basin [7]. Methanogens and sulphate reducers can co-exist and compete for resources, although sulphate reducers have a thermodynamic advantage compared to methanogens when using most electron donors, with the exception of when they are used with acetate. Thus, acetotrophic methanogens can be very active in the presence of sulphate [19]. Some studies [20,21] have shown that sulphate-induced inhibition of methanogenesis is associated with the ratio of grams of organic carbon (COD) that the microorganisms can oxidize to grams of sulphate. At low sulphate, when the COD/sulphate ratio is>10, sulphate reduction is minimal, and methanogenesis is unaffected [20], while at COD/sulphate ratios <1, the sulphate reducers can outcompete the methanogens [21]. Hence, the COD to sulphate ratio in our studies was likely >10.

The “intrinsic” methanogenic activity (Figure 1A and Table 2) was the highest with the MFT of Pond 7, followed by Pond 2/3 (5 m) and then Pond 2/3 (15 m). The order of the two MFT samples from Pond 2/3 correlates with the age of the tailings, with the higher rate occurring with the less dense, i.e., younger, MFT and the lower rate with the older, deeper MFT. A similar trend was found by Siddique et al. [22] with unamended MFT from a different OSTP, the Mildred Lake Settling Basin (MLSB), in which a sample taken at 6 m produced more methane than a sample taken at 31 m. The “age” of the material can reflect differences such as the microbial population and chemical composition.

Naptha (0.2 to 1.0% wt/vol) was added to the MFT samples under methanogenic conditions at concentrations consistent with those discharged into the tailing ponds (Small et al. 2015) [23]. No other ammendments were added. The results for the MFT of Pond 2/3 (5 m) (Figure 1B) were similar in all of the pond samples. With the addition of 0.2% (wt/vol) naphtha, CH_4_ production increased above the “intrinsic” rate (Figure 1B; note the difference in *y*-axis scale on Figure 1A,B). However, adding more naphtha (0.5 and 1.0% (wt/vol)) did not substantially increase methane production. There appeared to be an initial lag phase, with a rate of about 4.6 ± 1.4 µmole CH_4_ per 100 mL of MFT per day in the first 32 days followed by a higher rate of ~20.5 ± 2.5 µmole CH_4_ per 100 mL of MFT per day. However, we believe that the slower rate was not a true lag phase since nitrate-reducing and sulphate-reducing activities were typically evident within 5 days (Figures 4 and 5), and these samples were already methanogenic, as seen in Figure 1A. The change from the slower to the faster rate of methane production may be due to a shift in the metabolism from acetotrophic to hydrogenotrophic methanogens. Acetotrophic methanogens use acetate fermentatively as the electron donor and electron acceptor (CH_3_COOH → CH_4_ + CO_2_) [24], so they derive less energy, and their growth and methane production rates are slower. On the other hand, hydrogenotrophic methanogens use CO_2_ as the electron acceptor and H_2_ as the electron donor (CO_2_ + 4H_2_ → CH_4_ + 2H_2_O) [24] to generate more energy, resulting in higher growth and methane production rates.

The rate of methane production should have increased with increasing naphtha concentration if the cultures were carbon-limited. Several studies [7,10,11,25] that have added 1:1 MFT to a methanogenic medium have found that the rate of methane production approximated to the first order with respect to diluent components. Since we found no correlation with the amount of naphtha, one possible explanation is that nutrients such as a NH_4_^+^ or PO_4_^3-^ may limit the rate of methane production when the naphtha increases, especially since phosphate was below detectable levels and ammonium was low in the “as received” pore water from the MFT samples (Table 1).

**Figure 2:**
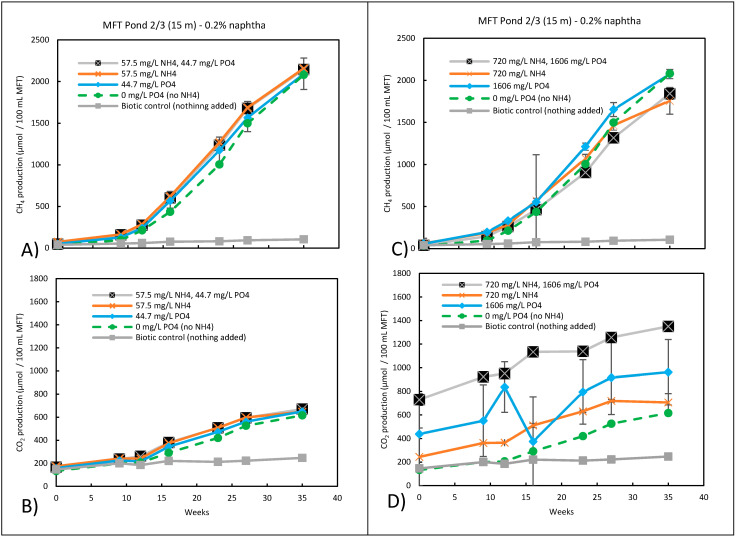
(**A**) CH_4_ and (**B**) CO_2_ production of duplicate microcosms for MFT from Pond 2/3 at 15 m amended with 0.2% naphtha and low concentrations of NH_4_^+^ and/or PO_4_^3−^. (**C**) CH_4_ and (**D**) CO_2_ production of duplicate microcosms for MFT from Pond 2/3 at 15 m amended with 0.2% naphtha and high concentrations of NH_4_^+^ and/or PO_4_^3−^. After naphtha addition, microcosms were mixed for 30–60 min on a rotary shaker then incubated in the dark. Error bars have been inserted, and where not visible, the error was too small to be seen.

To assess the effect of ammonium and phosphate in a reasonable timeframe, a small amount of naphtha (0.2% wt/vol) was added to enhance methanogenic activity. High ammonium and phosphate concentrations were based on the methanogenic medium of Fedorak and Hrudey [6], and low concentrations were based on Takashima and Speece [26] for the minimum requirements for methanogenesis. The results in Figure 2 for the Pond 2/3 (15 m) MFTs are representative of all of the pond samples. With the addition of 0.2% naptha alone (biotic control), methane production was significantly higher than when nothing was added (Figure 2A,C) using one-way ANOVA with Dunn’s post hoc test (*p* < 0.05). However, adding NH_4_^+^ or PO_4_^3−^ alone or in combination at low (57.5 mg/mL NH_4_^+^ and 44.7 mg/L PO_4_^3−^) or high (720 mg/mL NH_4_^+^ and 1606 mg/L PO_4_^3−^) concentrations resulted in similar methane production as when only naphtha was added. CO_2_ production at the lower concentration of NH_4_^+^ and PO_4_^3−^ followed a similar trend to CH_4_ (Figure 2B). The higher CO_2_ measured at t = 0 when nutrient levels were higher (Figure 2B,D) clearly indicates that abiotic processes were involved. For example, upon the addition of 286 or 1404 mg/L PO_4_^3−^ to Pond 7 MFT, the pH decreased from 7.95 to 7.30 and 7.03, respectively. This change in pH would have shifted the CO_2_ equilibrium between what is dissolved in water, what is present in the gas phase, and what is trapped as bicarbonate (~1900 mg/L) to release abiotic CO_2_. Over time, the equilbrium can be further affected by biologically produced and biologically consumed CO_2_, making the interpretation of the CO_2_ data difficult with any pH change.

### 3.3. Sulfidogenic Metabolism

**Figure 3:**
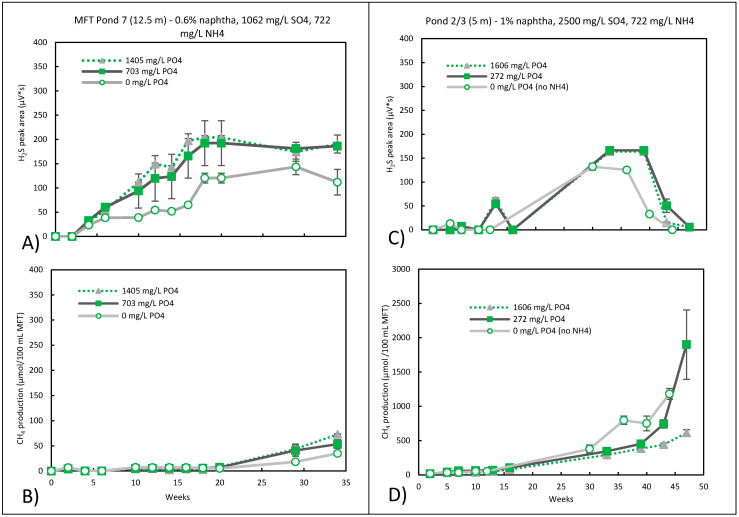
The effect of NH_4_^+^ and PO_4_^3-^ on (**A**) H_2_S and (**B**) CH_4_ production by Pond 7 (12.5 m) MFT and (**C**) H_2_S and (**D**) CH_4_ production by Pond 2/3 (5 m) under sulphate-reducing conditions. Measured sulphate concentrations in the pore waters of Pond 7 at week 0 was ~645 mg/L, which decreased to below detectable levels by week 34. For Pond 2/3 (5 m) at week 0, it was ~1400 mg/L, decreasing to ~24 mg/L by week 13, and was below detectable levels by week 44. Microcosms were setup in duplicate, error bars were inserted, and where not visible, the error was too small to be seen.

Sulfidogenic conditions were created by adding 1062 and 2500 mg/L of SO_4_^2−^ to MFT from Pond 7 (12.5 m) and Pond 2/3 (5 m), respectively, and sulphate reduction was confirmed by H_2_S production in the gas phase (Figure 3A,C). We relied on gas instead of pore water analysis to routinely track microbial activity as frequent water phase analyses were not practical because they required a large sample volume due to the high solid content. In the Pond 7 MFT, methane production was inhibited, and there was an increase in sulphate reduction with the addition of ammonium and phosphate compared to ammonium alone, but sulphate reduction did not increase with increasing phosphate concentration (Figure 3A,B). It was only with sulphate and no ammonium nor phosphate added to the MFT of Pond 2/3 (5 m) that methane production was inhibited, and sulphate-reducing activity was evident with a small amount of H_2_S production at day 5 (Figure 3C,D). With the addition of ammonium and phosphate, sulphate reduction was somewhat enhanced. Although the experiments were set up slightly differently, the data consistently showed that there was some enhancement in terms of sulphate reduction with the addition of ammonium and/or phosphate. In both MFT samples, methane production was inhibited for approximately the first 20 weeks, regardless of the amount of sulphate, ammonium, or phosphate that was added (Figure 3B,D).

There were a few notable differences in the results of the two pond samples. The H_2_S produced in the Pond 7 microcosms persisted (Figure 3A), with methanogenesis slowly resuming at a low level around week 30–33 (Figure 3B). On the other hand, although H_2_S was produced at a similar level in Pond 2/3 MFT (5 m), its disappearance began after 36–39 weeks of incubation (Figure 3C). Secondly, methanogenesis resumed at a higher rate in Pond 2/3 (5 m), even when H_2_S levels were still high but increased even further as the H_2_S disappeared (Figure 3D). Sulphate itself is not considered inhibitory to methanogens, but H_2_S is [27]. This may explain why the resumption of methanogenesis was so much slower in Pond 7, in which H_2_S persisted, compared to Pond 2/3 (5 m). The disappearance of H_2_S from the Pond 2/3 microcosms could be due to abiotic and/or biotic processes. H_2_S is very reactive and can react with metal ions to precipitate as metal sulphides. These black precipitates were not readily visible because the microcosm contents were obscured by bitumen. The chemical oxidation of H_2_S with oxygen or nitrate may also occur [28], but there was no oxygen or nitrate present. Although some anaerobic, autotrophic archaea can oxidize H_2_S with CO_2_ as the electron acceptor to obtain energy and support growth [29], H_2_S removal was likely to be primarily abiotic [30].

### 3.4. Nitrate Reduction

**Figure 4:**
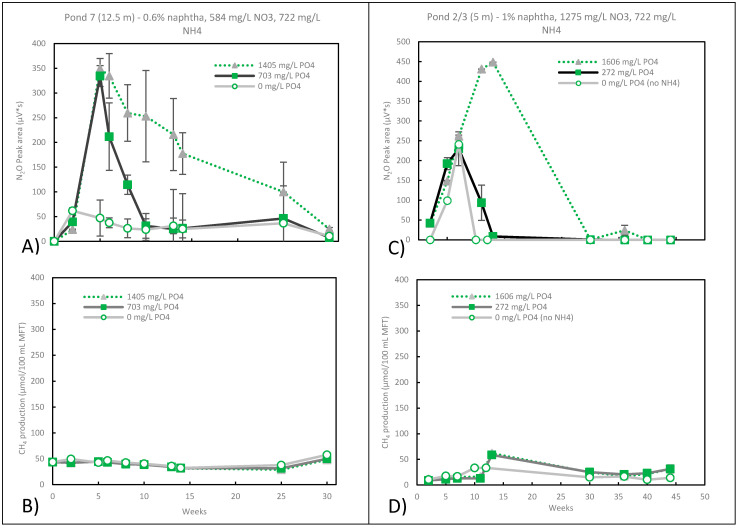
The effect of NH_4_^+^ and PO_4_^3-^ on (**A**) N_2_O and (**B**) CH_4_ production by Pond 7 (12.5 m) MFT and (**C**) N_2_O and (**D**) CH_4_ production by Pond 2/3 (5 m) under nitrate-reducing conditions. Measured nitrate concentrations in the pore waters of Pond 7 at week 0 were ~645 mg/L, which decreased to below detectable levels by week 34. For Pond 2/3 (5 m) at week 0, they were ~1400 mg/L, decreasing to ~24 mg/L by week 13 and were below detectable levels by week 44. Microcosms were setup in duplicate, error bars were inserted, and where not visible, the error was too small to be seen.

The pore water of the as received MFT from Pond 7 at 12.5 m and Pond 2/3 at 5 m had no detectable levels of nitrate (Table 1). Similar to the studies under sulphate-reducing conditions, either 0.6 or 1% (wt/vol) naphtha was added with ammonium and the increasing concentrations of phosphate. Methanogenesis was inhibited, and nitrate-reducing conditions were achieved by adding 584 and 1275 mg/L of NO_3_^−^ to the MFT of Pond 7 and Pond 2/3 (5 m), respectively (Figure 4A–D). Nitrate can be reduced through a series of intermediates (nitrate (NO_3_^−^) → nitrite (NO_2_^−^) → nitric oxide NO → nitrous oxide N_2_O → nitrogen gas (N_2_)) to nitrogen gas. Nitrate reduction was confirmed by the gas-phase evolution of N_2_O, which was observed as early as day 2 with the Pond 7 MFT, with the amount of N_2_O produced increasing with the increasing phosphate.

The addition of ammonium and phosphate was not required for nitrate reduction to occur in the Pond 2/3 (5 m) MFT (Figure 4C), and adding 722 mg/L ammonium and 272 mg/L phosphate resulted in a similar level of nitrate reduction. However, increasing the amount of phosphate (1606 mg/L) resulted in sustained N_2_O production over a longer period. Similar to Pond 2/3 (5 m), with the Pond 7 MFT, there was increasing nitrate-reducing activity w when the phosphate concentration increased (Figure 4A). N_2_O accumulation (Figure 4A,C) indicates a bottleneck at N_2_O → N_2_. However, the N_2_O reductase was active, and eventually, all of the N_2_O was reduced. This indicates that the N_2_O reductase had a low level of activity and/or its activity may have been inhibited by one or more NO_x_^−^ intermediates. NO_2_^−^ has been frequently associated with N_2_O accumulation [31,32], and free nitrous acid (HNO_2_) has also been linked to the inhibition of N_2_O reductase since inhibition depends on pH [33].

In both sets of microcosms, the t = 0 nitrate levels were a little higher than the amount that was added, and by the final time point, the nitrate was at non-detectable levels. At an intermediate time point for Pond 2/3 (5 m) at week 13, the nitrate level was ~24 mg/L, decreasing from an initial value of ~1400 mg/L for all samples. This was at the peak N_2_O at the highest phosphate concentration (Figure 4C). Based on the rate of nitrate reduction in the first 13 weeks, it is likely that all of the nitrate was gone shortly after. However, methanogenesis continued to be inhibited from weeks 13 to 44, when there was likely no nitrate and low N_2_O levels at the lower phosphate concentrations (Figure 4C). Other studies have shown that the products of nitrate reduction and not nitrate were more effective at inhibiting methanogenesis [34,35]. Nitrate had the weakest inhibition on methanogenesis by *Methanosarcina barkeri* and *Methanobacterium bryantii,* while N_2_O and NO_2_^−^ had an intermediate effect, and NO had the strongest inhibition [34]. These inhibitions were found to be reversible or irreversible depending on the microorganism and the concentration of the inhibitor.

The release of N_2_O from tailing ponds is not desirable, as it is a more potent greenhouse gas than methane. Methane has a global warming potential (GWP) of 28–36 over the next 100 years, while the GWP of N_2_O has a global warming potential of 265–298 over the next 100 years [5]. In our studies, which were conducted in a closed environment, the N_2_O was eventually totally consumed and was reduced to nitrogen gas. The conditions that best inhibited methanogenesis and minimized N_2_O production would be the addition of nitrate without ammonium and/or phosphate (Figure 4A,C).

Although there was some increasing microbial activity with increasing nutrient concentrations under nitrate- and sulphate-reducing conditions, this was not evident under methanogenic conditions. In this complex MFT environment, there are other factors that may also affect degradation rates, such as the poor bioavailability of naphtha. There was a significant amount of visible bitumen in all pond samples, with the most being observed in Pond 2/3 (5 m). It is likely that a majority of the naphtha partitioned into the residual bitumen, limiting naphtha’s bioavailability [36] and hence its rate of biodegradation. In such a case, the naphtha degradation rate and hence its microbial activity would be limited either by the partitioning of naphtha into the aqueous phase where naphtha could be consumed by the naphtha-degraders [37] or by the hydrocarbon-degrading bacteria accessing naphtha by being in direct physical contact at the bitumen/aqueous phase interface [37,38]. Thus, even though ammonium and phosphate may be limiting, in the presence of bitumen, the bioavailability of naphtha is an even greater issue and may account for the results seen in Figure 1B. From the tested conditions, the addition of sulphate was also less effective at inhibiting methanogenesis than nitrate was. This may be related to the ease with which H_2_S can be chemically removed via precipitation with metals.

### 3.5. Potential Fate of Ammonium and Phosphate

**Figure 5:**
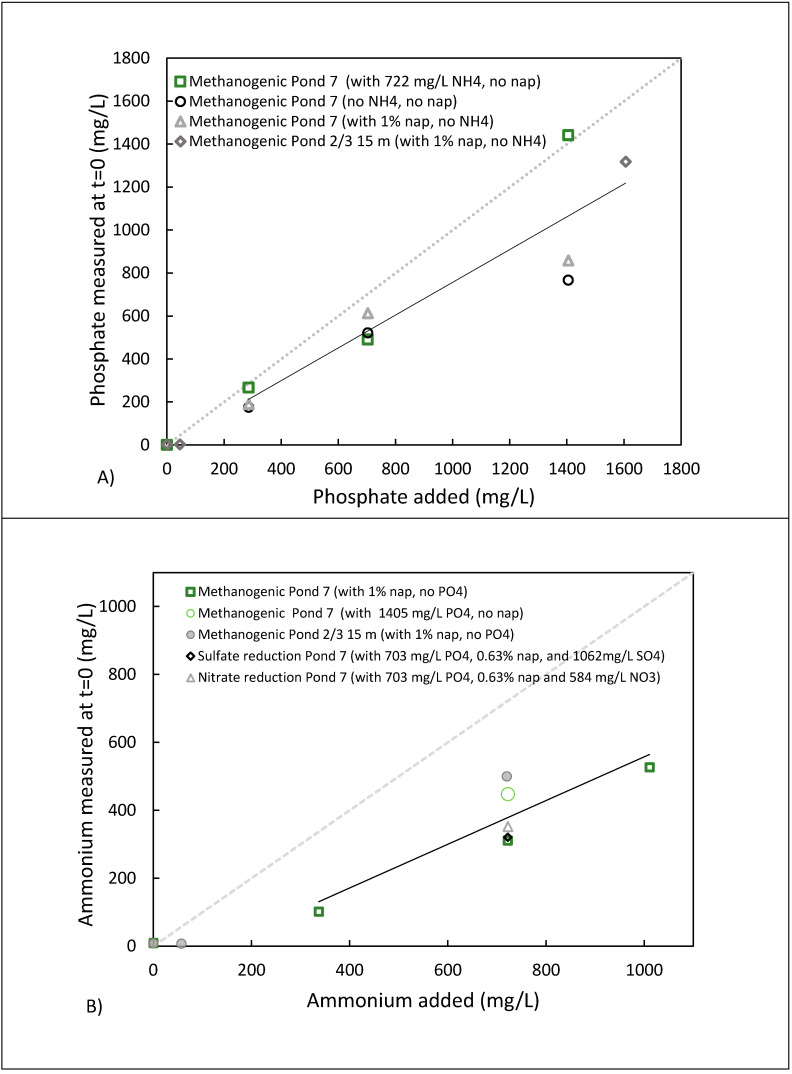
Plots showing the amount of (**A**) PO_4_^3−^ and (**B**) NH_4_^+^ added to MFT samples and the “initial” concentration measured in the pore water. After phosphate and/or ammonium were added, the pore water was separated via centrifugation within a maximum of 6 h of experimental setup.

When concentrations of ammonium, phosphate, sulphate, or nitrate were prepared in just water, the measured aqueous concentration was close to the prepared concentrations and did not vary with time. However, after phosphate and/or ammonium were added to the MFT samples, the concentrations measured in the pore water recovered via centrifugation within 6 h of the experimental setup (i.e., “initial” concentrations) were much lower. For example, when 44.7 or 272 mg/L of phosphate was added, there was no detectable phosphate in the t = 0 samples, indicating a rapid loss. When >272 mg/L of phosphate was added, the t = 0 measurements ranged from 25–35% less than the amount added (Figure 5A). At low ammonium concentrations, there was almost total removal (e.g., ~80% loss at 57 mg/L) and 30–45% loss at higher concentrations (Figure 5B). Similar ammonium losses were observed whether nitrate or sulphate were also present.

This could explain why adding ammonium and phosphate did not affect methanogenesis at lower concentrations and only somewhat affected sulphate or nitrate reduction at the higher concentrations. It is also likely that the methanogens may not need ammonium, as it was recently reported that some methanogens may fix N_2_ in marine hyperthermophilic and thermophilic cultures [39], as determined in enrichment cultures from an Albian MFT [15] and in soils in the Florida Everglades [40]. However, further work is required to determine whether nitrogen-fixing methanogens are active in our studies. Higher ammonium concentrations may be inhibitory to some of these cultures and may have no effect on others [41].

The disappearance of phosphate and ammonium may be due to mineral precipitation or sorption onto the MFT matrix. To check for potential precipitates, after being mixed with ammonium and phosphate, the dried MFT solids were analyzed by X-ray diffraction. There was no evidence of precipitates such as struvite (NH₄MgPO₄·6H₂O), so if any precipitates were formed, they were below detectable levels.

The major mineral content of Pond 7 includes 27–31% quartz (SiO_2_), 32–43% kaolinite (Al_2_Si_2_O_5_(OH)_4_), ~20% muscovite ((KF)_2_(Al_2_O_2_)_2_(SiO_2_)_2_), 2–4% siderite (FeCO_3_), 0.5–1.0% calcite (CaCO_3_), and feldspars (Per. Comm. E. Hollander, Suncor) [4]. Many of these components have potential for sorption. The sorption of anions such as phosphate onto clays [42,43,44,45,46,47,48] has been extensively studied. While the sorption of cations such as ammonium onto clays [49,50] and either ammonium or phosphate onto quartz [51,52,53], muscovite [53], calcite [52,54], or iron minerals (e.g., geothitie) [43,48,55] have been demonstrated but have been less well studied. However, we were unable to find any published studies with MFT.

Clays such as kaolinite can sorb both cations and anions and have a higher cationic than anionic exchange capacity. This is reflected in the higher initial loss of NH_4_^+^ compared to PO_4_^3-^ (Figure 5). Cations such as NH_4_^+^ can sorb via two mechanisms. One mechanism occurs via the permanent negative charge on the basal planes of the clay surface whereby the surface cations (e.g., Al^3+^ or Si^4+^) can be displaced by a species with a lower charge, e.g., NH_4_^+^, and hence does not depend on pH or ionic strength. However, the other mechanism depends on pH and ionic strength as the cations are sorbed to the surface by electrostatic interaction with the OH^−^ groups arising from the deprotonation of the aluminol and silanol groups along the variable charged edges [56]. The pore water of the MFT contains high concentrations of competing cations (e.g., Na^+^, K^+^, and Ca^2+^) that have been shown to decrease the sorption of NH_4_^+^ onto montmorillonite [57].

Anion adsorption occurs through the second mechanism and hence strongly depends on pH. However, adsorption of PO_4_^3-^ is thought to be more complex and is thought to be linked to Al sites and may also involve multilayer adsorption (including penetration into the inter-lamellar spaces) and/or surface precipitation [42]. The latter is a very slow process that affects the aqueous concentration of phosphate after several days to weeks [42]. Thus, the rapid, early disappearance of ammonium and phosphate observed upon their addition to MFT is consistent with adsorption. Multilayer adsorption can occur at higher phosphate loadings as it moves into the inter-lamellar spaces and into amorphous regions of the clay surface [58]. While a significant amount of the MFT is kaolinite, there are other layered phyllosilicate minerals such as muscovite that comprise a substantial amount of the solids. The dominant charged groups in the feldspars are aluminol and silanol, which are also found in kaolinite and muscovite. These minerals have similar surface reactions in aqueous systems [59] and may be involved in the sorption of NH_4_^+^ and PO_4_^3−^.

When Gerard (2016) [42] and Wei et al. (2014) [48] compared the binding of phosphate to a clayey (kaolinite) vs. sandy soil (i.e quartz silicates), they found that sorption onto a sandy soil was minor. For example, Gerard (2016) [42] calculated that a maximum of 15–50% phosphate would bind to a clayey soil compared to 0.5–3% in a sandy soil. Thus, in MFT, a much higher portion of anions that are similar to phosphate can be expected to bind to kaolinite compared to the quartz component.

Assuming that there were no other limitations, sorption onto the mineral components of MFT and non-detectable levels of phosphate in the pore water would ultimately lead to microbial activity being limited by the desorption equilibrium between nutrients sorbed on the mineral surface and the aqueous phase and their microbial utilization. Microbial activity in OSTPs appears to be controlled by multiple nutrient limitations. Apart from the terminal electron acceptor, it could include carbon, ammonium, and phosphate limitations.

## 4. Conclusions

The rate of methane production in unamended MFT samples correlated with the age of the sample such that it was the highest in the least dense (i.e., most recently discharged) MFT and was the lowest in the MFT sample that was most dense (i.e., the oldest). The addition of a carbon substrate increased methane production, but there was no correlation when the naphtha concentration was increased. Although non-detectable levels of phosphate and low ammonium indicated that these nutrients were potentially limiting microbial activity, their addition had no significant effect on methanogenesis but did somewhat enhance sulphate and nitrate reduction. This might be due to a combination of the loss of ammonium and phosphate via adsorption onto MFT minerals and the poor bioavailability of naphtha, which may have partitioned into the bitumen phase.

While both sulphate- and nitrate-reducing conditions inhibited methanogenesis, nitrate better sustained the inhibition for a longer duration. Under nitrate-reducing conditions, N_2_O, a potent greenhouse gas was produced but was eventually consumed. Its production was the lowest without the addition of ammonium or phosphate and had a similar level of inhibition as when more N_2_O was produced. The lower effectiveness of sulphate to inhibit methanogenesis may be associated with a lower effectiveness of H_2_S as an inhibitor compared to the NO_x_^−^ intermediates and the ease by which H_2_S can be abiotically removed. However, N_2_O is a potent greenhouse gas. We have demonstrated that multiple factors, such as the availability of carbon, nitrogen, and phosphorous substrates, simultaneously affect microbial activity in OSTPs.

## Figures and Tables

**Table 1:** Characterization of three MFT samples. ^a^ Per. Comm. E. Hollander, Suncor [4].

Parameter	Pond 2/3 at 5 m	Pond 2/3 at 15 m	Pond 7 at 12.5 m
PO_4_^3−^ (mg/L)	<3.1	<3.1	<3.1
NH_4_^+^ (mg/L)	20.8 ± 0.5	35.5 ± 0.7	6.4 ± 2.4
SO_4_^2−^ (mg/L)	20	100	< 1.0
NO_3_^−^ (mg/L)	<0.5	<0.5	<0.5
NO_2_^−^ (mg/L)	<0.33	<0.33	<0.33
Eh (mV)	−123	−153	−200 ± 40
Naphthenic Acid (μg/L) ^a^	15,100	54,700	27,600
pH	8.41 ± 0.1	8.39 ± 0.1	8.11 ± 0.4
Alkalinity, Bicarbonate (mg/L) ^a^	2100	1900	1900
DOC (mg/L) ^a^	56	24	46
Hardness (mg CaCO_3_/L) ^a^	240	180	160
Total dried solids (g/L)	743 ± 11.47	1000 ± 3.30	415 ± 4.77

**Table 2:** Methanogenic rates in unamended (i.e., nothing added) MFT microcosms calculated using the data from Figure 1A. Values are the average of at least two measurements, except for *, which is a single analysis or NM (not measured).

Unamended MFT (Nothing Added)	pH		Redox (mV)		CH_4_ Generation Rate (µmole/100 mL MFT/Day)
	Initial	Final	Initial	Final	
Pond 7 at 12.5 m	7.89 ± 0.1	7.82 ± 0.1	−136 ± 11	−247 ± 4	0.72 ± 0.12
Pond 2/3 at 5 m	7.13 ± 0.3	7.84 ± 0.1	−120 ± 2	−129 ± 12	0.56 ± 0.04
Pond 2/3 at 15 m	7.39 *	NM	−155 *	NM	0.30 ± 0.01

## Data Availability

The data presented in this study are available within the manuscript.
